# Enhancement of liver mitochondrial complex I and energy metabolism induced by enteritis: The key role of gut microbiota derived endotoxins

**DOI:** 10.3389/fimmu.2022.981917

**Published:** 2022-08-23

**Authors:** Lele Fu, Haokun Liu, Wen Chen, Jamie Marie Hooft, Margareth Øverland, Wanjie Cai, Dong Han, Xiaoming Zhu, Yunxia Yang, Junyan Jin, Shouqi Xie

**Affiliations:** ^1^ State Key Laboratory of Fresh Water Ecology and Biotechnology, Institute of Hydrobiology, Chinese Academy of Sciences, Wuhan, China; ^2^ College of Advanced Agricultural Sciences, University of Chinese Academy of Sciences, Beijing, China; ^3^ Department of Animal and Aquacultural Sciences, Faculty of Biosciences, Norwegian University of Life Sciences, Ås, Norway; ^4^ Hubei Engineering Research Center for Aquatic Animal Nutrition and Feed, Wuhan, China; ^5^ The Innovative Academy of Seed Design, Chinese Academy of Sciences, Wuhan, China

**Keywords:** intestinal inflammation, intestinal permeability, endotoxin, metabolism, mitochondrial complex I

## Abstract

Inflammation is an energy-intensive process and the liver is a key organ in energy regulation. Since the intestine and liver exchange nutrients and metabolites, enteritis can affect the liver. To investigate the correlation between enteritis and liver metabolism, we developed an intestinal inflammation model with concentration-dependent 2,4,6-trinitrobenzene sulfonic acid (TNBS) in gibel carp (*Carassius gibelio*). The results showed the dysregulation of intestinal tight junction, increased permeability of the gut barrier, and apoptosis of epithelial cells during the development of enteritis. The liver metabolome was analyzed by LC-MS and the live respiration was determined using Oxygraph-2k. The results showed that glycolysis, the TCA cycle and pyrimidine metabolism were affected by intestinal inflammation. In particular, the activity of hepatic mitochondrial respiratory chain complex I was significantly increased. Structure and abundance changes of gut microbiota were analyzed by 16S rRNA sequencing analysis. Pathogenic bacteria in the intestine, as well as plasma LPS, increased significantly. Using a liver cell line, we verified that the dysfunctional metabolism of the liver is related to the dislocation of LPS. All results imply the existence of a connection between enteritis and liver metabolism in gibel carp, and the gut microbiome plays a critical role in this process.

## Introduction

The gastrointestinal tract is continuously exposed to numerous bacteria as well as food-derived and environmental toxins (1), potentially leading to inflammatory bowel diseases (IBD) in humans (2, 3) and colitis in animals (4–6). IBD presents with abdominal pain, fever, and clinical signs of bowel obstruction or diarrhea with passage of blood or mucus, or both. Enteric diseases in commercial animals contribute to losses in productivity and increased mortality. The dysregulation of intestinal permeability, tight junction, immune function, and apoptosis has shown to be involved in the development of enteritis (7). As an important part of the intestinal immune barrier, the gut microbiota is crucial for maintaining the host’s health (8). The development of intestinal inflammation is accompanied by dysbiosis of intestinal microbiota. The leakiness and dysregulated permeability of the gut not only enables bacteria to invade the host, but also results in the infiltration of macrophages and inflammation. In response, the body produces cytokines and other mediators, effectively launching a systemic inflammatory response (9). Thus, enteritis is a systemic disease that not only affects the gastrointestinal tract, but also impacts the extraintestinal organs. The effects of intestinal microbiota and microbial metabolites on other organs during enteritis is therefore worthy of future study.

Dysregulated energy homeostasis has been observed to be implicated in the pathogenesis of enteritis (10). Mitochondrial oxidative phosphorylation is the main source of cellular adenosine 5’-triphosphate (ATP). A study in ulcerative colitis patients found that complex II and III were decreased by around 50-60% in the colonic mucosa (11). Other studies in mice showed that increasing mitochondrial ATP synthesis in intestinal epithelial cells could relieve colitis (10). Therefore, the regulation of energy metabolism is anticipated as a promising strategy for counteracting enteritis. As a main organ of metabolism, the liver is exposed to intestinal-derived substances including nutrients and bacteria. Some studies have found that some IBD patients or animals with enteritis exhibited liver diseases, such as non-alcoholic fatty liver disease (NAFLD) and metabolic disturbances (12, 13). However, the mechanisms which alter mitochondrial activity in the liver during intestinal inflammation are still unknown.

In the present study, we speculated that intestinal inflammation may impact the gut microbiota, thereby affecting the gut-liver axis and causing a change in liver mitochondrial metabolism in response to inflammation. To test this hypothesis, we developed an intestinal inflammation model using gibel carp (*Carassius gibelio*). The structure and abundance of changes in the gut microbiota were analyzed by 16S rRNA sequencing analysis. Liver metabolome was analyzed by LC-MS, and we quantified mitochondrial activity in the liver by Oxygraph-2k. We also treated a liver cell line with LPS in order to verify that the dysfunction of energy metabolism during intestinal inflammation is related to the gut microbiota.

## Materials and methods

### Animal ethics

All experiments were conducted under the Guidance of the Care and Use of Laboratory Animals in China. This study was approved by the ethics committee of the Institute of Hydrobiology, Chinese Academy of Sciences.

### TNBS model of fish enteritis

Gibel carp were obtained from the Institute of Hydrobiology, Chinese Academy of Sciences (Wuhan, China). The fish were cultured in an indoor recirculating aquaculture system throughout the acclimation and experimental periods (22.5-29.5°C, pH 7.0-7.5, dissolved oxygen >5 mg/L, ammonia nitrogen concentration <0.5 mg/L, light intensity 2.72-2.93 μmol/m^2^/s, 12h inverted light/dark cycle). During the acclimation period, fish were fed the control diet three times (8:00, 14:00, and 19:00) per day.

The administration of TNBS (Sigma-Aldrich) was performed by gavage using a lavage tube (OD 1.00 mm). After a 24h period of fasting, the fishes were anesthetized with MS-222 (50 mg/L, Sigma, Saint Louis, Missouri, USA) and a TNBS-ethanol solution (50% *v/v*) was administered at a dose of 75 μL or 125 μL/kg body weight (TNBS 75 μL/kg; TNBS 125 μL/kg). The control groups were treated with the same volume of 0.68% stroke-physiological saline solution (SS 75 μL/kg; SS 125 μL/kg).

Fish were anesthetized for sampling after 24h and blood samples were taken from the caudal vein, centrifuged at 3,000 *g* (5 min, 4°C), and stored at -80°C until further analyses. Tissue samples were collected and stored at -80°C for further analyses including biochemical assays, gene expression assessment, and metabolomic analysis. A small section of each liver and mid-gut was fixed in 4% paraformaldehyde solution for histological analysis. Another portion of the mid-gut was stored in 2.5% glutaraldehyde solution for ultrastructural observation. In addition, another 7 fish in the TNBS 125 μL/kg and SS 125 μL/kg groups were sampled for testing intestinal epithelial resistance by the Ussing Chamber. The liver was collected for analysis of respiration by Oxygraph-2k immediately after sampling.

### Histological analyses

Gross pictures of the whole intestine from all groups of fish were taken immediately following dissection (DC-GF10K, Panasonic). Hematoxylin-eosin (H&E) staining of the intestine and liver samples was performed by Powerful Biology Co. (Wuhan, China), and photographed using light microscopy (Leica DM2500, Leica, Solms, Germany). Transmission electron microscopy (TEM) observation of the intestine was conducted as described in Liu et al. (14).

A terminal deoxynucleotidyl transferase-mediated dUTP-biotin nick end labeling (TUNEL) technique was applied to verify apoptosis in the liver. The TUNEL and DAPI (4’, 6-diamidino-2-phenylindole) double staining of the liver was performed by Powerful Biology Co. (Wuhan, China) and slides were observed under a Tecnai transmission electron microscope.

### Ussing chamber experiments

The mucosal biopsies were immediately immersed in ice-cold Krebs-Ringer solution (117 mmol/L NaCl, 25 mmol/L NaHCO_3_, 1.2 mmol/L NaH_2_PO_4_, 2.5 mmol/L CaCl_2_, 1.2 mmol/L MgCl_2_, 4.7 mmol/L KCl_2_ and 11 mmol/L glucose), at pH 7.4 and gassed with 95% O_2_ and 5% CO_2_ at 25°C. The biopsied intestine was mounted in Ussing chambers (Beijing Kingtech Technology Co.) with an insert with a square area of 0.04 cm^2^, and each chamber was filled with 5 mL Krebs-Ringer solution which was maintained at 25°C and continuously oxygenated with 95% O_2_ and 5% CO_2_, and stirred by gas flow in the chambers as previously described (15). Through a short voltage pulse (5V), the epithelial resistance (Rep) is calculated by applied voltage with the current deflection.

### Biochemical assays

The plasma glucose, cholesterol, triglycerides, and non-esterified fatty acids (NEFAs) were analyzed using commercially available kits (Fujifilm, Wako Pure Chemical Corporation, Osaka, Japan). The plasma lipopolysaccharide (LPS) and diamine oxidase (DAO) (Jiangsu Meimian industrial Co. Ltd., China) were analyzed using an enzyme-linked immunosorbent assay method with a commercially available ELISA kit according to the manufacturer’s instructions. In addition, interleukin 1β (IL-1β), interleukin 6 (IL-6), interleukin 10 (IL-10), insulin and glucagon were analyzed using ELISA kits (Nanjing Jiancheng Bioengineering Institute, China). The total antioxidant capacity (T-AOC), superoxide dismutase (SOD) and reduced glutathione (GSH) were likewise analyzed using commercially available kits (Nanjing Jiancheng Bioengineering Institute, China).

### qPCR analysis

Total RNA was extracted and the quality was checked using 1.0% agarose electrophoresis. Subsequently, the mRNA quantitative reverse transcription-polymerase chain reaction (RT-PCR) was performed as previously described (16). The specific primers used for RT-PCR are referenced in previous studies (16) and the additional primers are listed in **Table 1**. The expression levels in the intestine were calculated as fold-change in expression relative to the housekeeping gene (*β-actin* and *ef-1α*).

**Table 1:** Sequences of primers applied for quantitative real-time PCR analysis in gibel carp.

Gene name	prime sequence	Product size (bp)	Gene bankaccession No.
Claudin 4	F: GCCGGTGTGATTTTCATCGTT	108	XM_026234420.1
	R: CTGTGCTTGGTTCAACAAGGG		
Interleukin-10 (*il-10*)	F: AGCCATGGGAGAGCTTGATA	227	XM_026275831.1
	R: ATGATGACGTGCAAGCGTT		
Apoptosis regulator Bcl-2 (*bcl2*)	F: AAAGGATGTACCAGCGCGAA	83	XM_026237836.1
	R: GGCTAAGAATCTGCGTTGCG		
Caspase 3 (*casp3*)	F: ATCATGACCAGGGTCAACCA	119	XM_026266756.1
	R: TACATCTCTTTGGTGAGCAT		
Endoplasmic reticulum oxidoreductase 1 alpha (*ero1α*)	F: ATGCCCAACACAAGCAACAC	129	XM_026242578.1
	R: TGACAACAGCGACCGAAAGT		
Peroxiredoxin (*prx*)	F: AGGTCATCGCTGCTTCCACCG	90	XM_026211451
	R: TGTTCATGGAGCCCAGGCCAC		
CNC homolog 1 (*bach1*)	F: TGGAGCGCAGGAGCTTTCGAG	98	XM_026282740
	R: AGTGGGGTTTGGTCGGCTGTG		
Superoxude dismutase (*sod*)	F: GTCCGCACTACAACCCTCAT	134	JQ776518.1
	R: GGTCACCATTTTATCCACAA		
Catalase (*cat*)	F: CTCCAACGGCAACTTCCCAT	102	JX477239.1
	R: CACACCTTAGTCAAATCAAA		
Heat shock protein 70 (*hsp70*)	F: CTCAACAAGAGCATCAACCCAG	155	JN006055.1
	R: ATGACTCCACCAGCCGTTTC		

### Liver respiration

The liver was excised from sacrificed fishes and precooled respiration medium was added to the tissues (0.05mM EGTA, 110 mM sucrose, 1g/L BSA, 20 mM HEPES, 10 mM KH_2_PO_4_, 20 Mm taurine, 60 mM K-lactobionic acid, and 3 mM MgCl_2_, pH7.1). A two-channel titration injection respirometer (Oxygraph-2k, Oroboros Instruments) was used to analyze liver respiratory function after homogenization (temperature was 28°C). The oxygen flux per tissue mass (pmol/(s*mg)) and oxygen concentration (μM) were recorded in real-time using Oroboros Datlab software (Oroboros Instruments). Routine respiration was measured when respiration was stabilized without any additives. After titration with malate (M, 2 mM) and pyruvate (P, 5 mM), the respiratory leak of the complex I was tested. The oxidative phosphorylation capacity of complex I (CI OXPHOS) was examined after adding 5 mM ADP. Succinate (10 mM) was titrated to measure OXPHOS of CI and complex II (CI&II OXPHOS). Then, stepwise addition of carbonylcyanide-*m*-chlorophenylhydrazone (CCCP, 2 μM) was carried out in order to obtain the maximal uncoupled respiratory capacity of the electron transfer system (CI&II ETS). Subsequently, the CII ETS was determined after adding rotenone (Rot, 0.5 μM). Finally, antimycin A (Ama, 2.5 μM) was added to evaluate the residual oxygen consumption (ROX). All substrates were dissolved in H_2_O and the inhibitors were dissolved in ethanol.

### 16S rRNA amplicon sequencing and bioinformatic analyses

Microbial genomic DNA was extracted using the E.Z.N.A.^®^ soil DNA Kit (Omega Bio-Tek, Norcross, GA, U.S.) and the quality was checked by agarose gel electrophoresis and NanoDrop 2000 UV-vis spectrophotometer (Thermo Scientific, Wilmington, USA). To amplify the hypervariable region V3-V4, the primer pairs 338F (5’-ACTCCTACGGGAGGCAGCAG-3’) and 806R (5’-GGACTACHVGGGTWTCTAAT-3’) were used. After extraction from 2% agarose gel, the PCR product was purified using the AxyPrep DNA Gel Extraction Kit (Axygen Biosciences, Union City, CA, USA) and quantified using Quantus™ Fluorometer (Promega, USA). The Illumina MiSeq sequencing was conducted by Shanghai Majorbio Bio-pharm Technology Co., Ltd and the data were analyzed using the online platform Majorbio Cloud Platform (www.majorbio.com).

### Cell culture and treatments

The NCTC1469 murine liver cell line (China Center for Type Culture Collection, Wuhan, China) was cultured in high-glucose Dulbecco’s modified Eagle’s medium (H-DMEM) (Biological Industries) supplemented with 10% fetal bovine serum (FBS) (Biological Industries), 100 units/mL penicillin, and 0.1mg/mL streptomycin (Biological Industries) at 37°C with humidified air and 5% CO_2_. The liver cells were plated at 1×10^7^ per well and treated with 1μg/mL lipopolysaccharides (LPS) (Sigma, O55:B5) or PBS (Biological Industries) for 24h. Subsequently, metabolomic analysis was conducted.

### Metabolome analysis of liver using LC-MS

The 50-milligram liver sample was placed into the 2-mL EP tube and 500 μL of pre-cooled extractant was added (70% methanol aqueous solution). After homogenizing, the sample was centrifuged at 1500 rpm for 5 min and then allowed to stand for 15 min on ice before being centrifuged again for 10 min at 12000 rpm. Subsequently, 200 μL of the supernatant was collected for analysis using an LC-ESI-MS/MS system (UPLC, Shim-peck UFLC SHIMADZU CBM30A system; MS, QTRAP^®^ System) which was equipped with electrospray ionization (ESI) Turbo Ion-Spray interface, operating in positive and negative ion mode and controlled by Analyst 1.6.3 software (Sciex). A specific set of multiple reaction monitoring (MRM) transitions was monitored for each period according to the metabolites eluted within this period.

All data were processed and normalized using MetaboAnalyst 5.0 (https://www.metaboanalyst.ca/). The samples were grouped using principal component analysis (PCA) and significantly up-regulated and down-regulated metabolites between groups were determined by variable importance in the projection (VIP) ≥ 1 and absolute Log_2_FC (fold change) ≥ 1.

### Metabolome analysis of cells using GC-MS

The 1×10^7^ cells were homogenized with 0.6 mL of cold 80% methanol and ground with a tissue-grinding machine. The samples were centrifuged at 13000 rpm for 20 min at 4°C after standing for 30 min at -20°C. Ribitol (15 μL) was added to the supernatant as an internal standard before drying with nitrogen at 45°C. The dried samples were derivatized by the addition of 40 μL of methoxyamine hydrochloride in pyridine and incubated for 90 min at 30°C. After that, 40 μL of N-Methyl-N-(trimethylsilyl)-trifluoroacetamide (MSTFA) (1% Trimethylchlorosilane) was added to each sample at 37°C for 30 min before centrifugation at 13000 rpm for 20 min at 4°C. The supernatant was transferred to an auto-sample vial for GC/MS analysis which was performed by the Center for Instrumental Analysis and Metrology (Institute of Hydrobiology, Chinese Academy of Science, Wuhan, China). All data were processed and normalized using MetaboAnalyst 5.0.

### Statistical analyses

Statistical comparisons were made among treatments using the statistical software SPSS 20.0 (SPSS Inc., Chicago, IL, USA). Student’s *t*-tests were conducted (unpaired, two-tailed). All values were expressed as the mean ± standard error (S.E.), and *P*<0.05 was considered significant.

## Results

### Establishment of intestinal inflammation in TNBS-treated gibel carp

**Figure 1:**
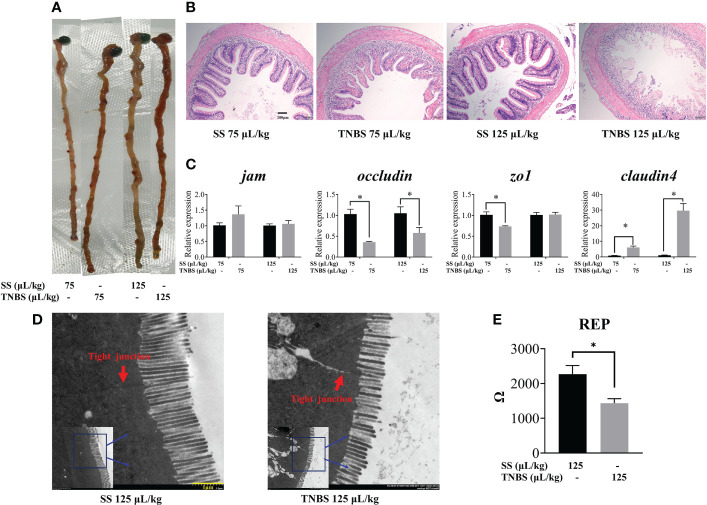
The effect of TNBS on the intestinal morphology and tight junction. **(A)** Gross picture of intestinal. **(B)** Each group’s representative microphotograph of H&E staining paraffin-embedded sections of gut was shown (scale bar: 100μm). **(C)** Relative mRNA levels of tight junction related genes. **(D)** 125μL/kg SS and 125μL/kg TNBS representative transmission electron microscope image of intestinal (scale bar: 1μm). Red arrows represent the tight junction. **(E)** Gut epithelial resistance of 125μL/kg SS and 125μL/kg TNBS. All data are expressed as mean ± S.E.M. (N = 6 at least). *P* value was calculated by Student’s *t* tests. *P< 0.05, compared with PBS groups.

Macroscopically, visible damage in TNBS-treated fishes was observed compared to the control group. As demonstrated by abnormal gross morphology and H&E staining (**Figures 1A, B**), TNBS-treatment induced dose-dependence gut congestion and erosion, and intestinal epithelial villi detachment and destruction.

TNBS significantly decreased intestinal barrier function. The expression of intestinal tight junction mRNA including *jam*, *zo1*, *occludin*, and *claudin4* is suggested to be representative of paracellular barrier integrity. Epithelial resistance (Rep) is determined by the permeability of tissue. As shown in **Figure 1C**, we found that the expression of *occludin* and *zo1* were significantly decreased in the 75 μL/kg TNBS treated group compared to the control, while *occludin* was significantly decreased in the 125 μL/kg TNBS treated group compared to the 125μL/kg SS group. Similar to the results of gene expression, the ultrastructural changes of the intestine (**Figure 1D**) revealed that tight junctions were significantly destructed in the 125 μL/kg TNBS group. The Rep also decreased significantly at this concentration of TNBS (**Figure 1E**), further confirming TNBS-induced increased permeability. Additionally, the expression of *claudin4* significantly increased in TNBS treated groups.

**Figure 2:**
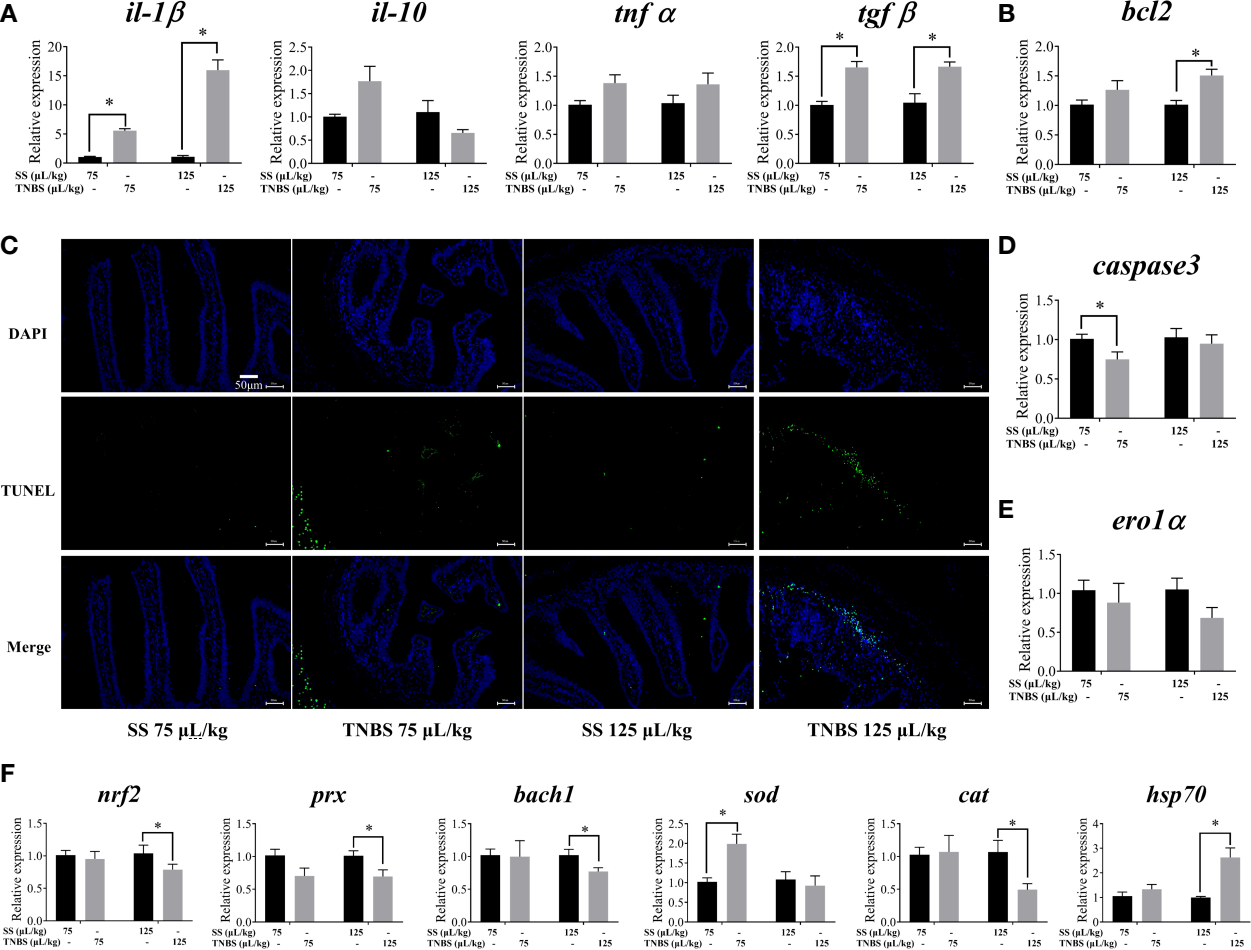
The effect of TNBS on the intestinal inflammation, apoptosis and oxidative damage. **(A)** mRNA levels of inflammatory cytokines. **(B, D, E)** Relative mRNA levels of apoptosis related genes. **(C)** Representative DAPI and TUNEL double staining of gut (scale bar: 50μm). **(F)** mRNA levels of oxidative-related genes. All data are expressed as mean ± S.E.M. (N = 6 at least). *P* value was calculated by Student’s t tests. *P< 0.05, compared with PBS groups.

Proinflammatory cytokines and apoptosis were also upregulated. Compared to the SS groups, there was higher expression of *il-1β* and *tgfβ* in TNBS-treated groups (**Figure 2A**). DAPI and TUNEL staining of the intestine indicated a greater degree of apoptosis in the TNBS-treated groups compared to the control groups (**Figure 2C**). In addition, 125 μL/kg TNBS significantly induced the transcription levels of *bcl2*, while *capsase3* was significantly downregulated in the 75 μL/kg TNBS group (**Figures 2B, D**). However, no significant difference in the expression of *ero1α* was observed (**Figure 2E**).

TNBS induced intestinal oxidative damage in gibel carp (**Figure 2F**). Exposure to 125 μL/kg TNBS significantly down-regulated the expression of *nrf2*, *prx*, *bach1* and *cat*, and up-regulated *hsp70*. Furthermore, 75 μL/kg TNBS significantly enhanced the transcription of *sod*.

### Dysbiosis of intestinal microbiota

**Figure 3:**
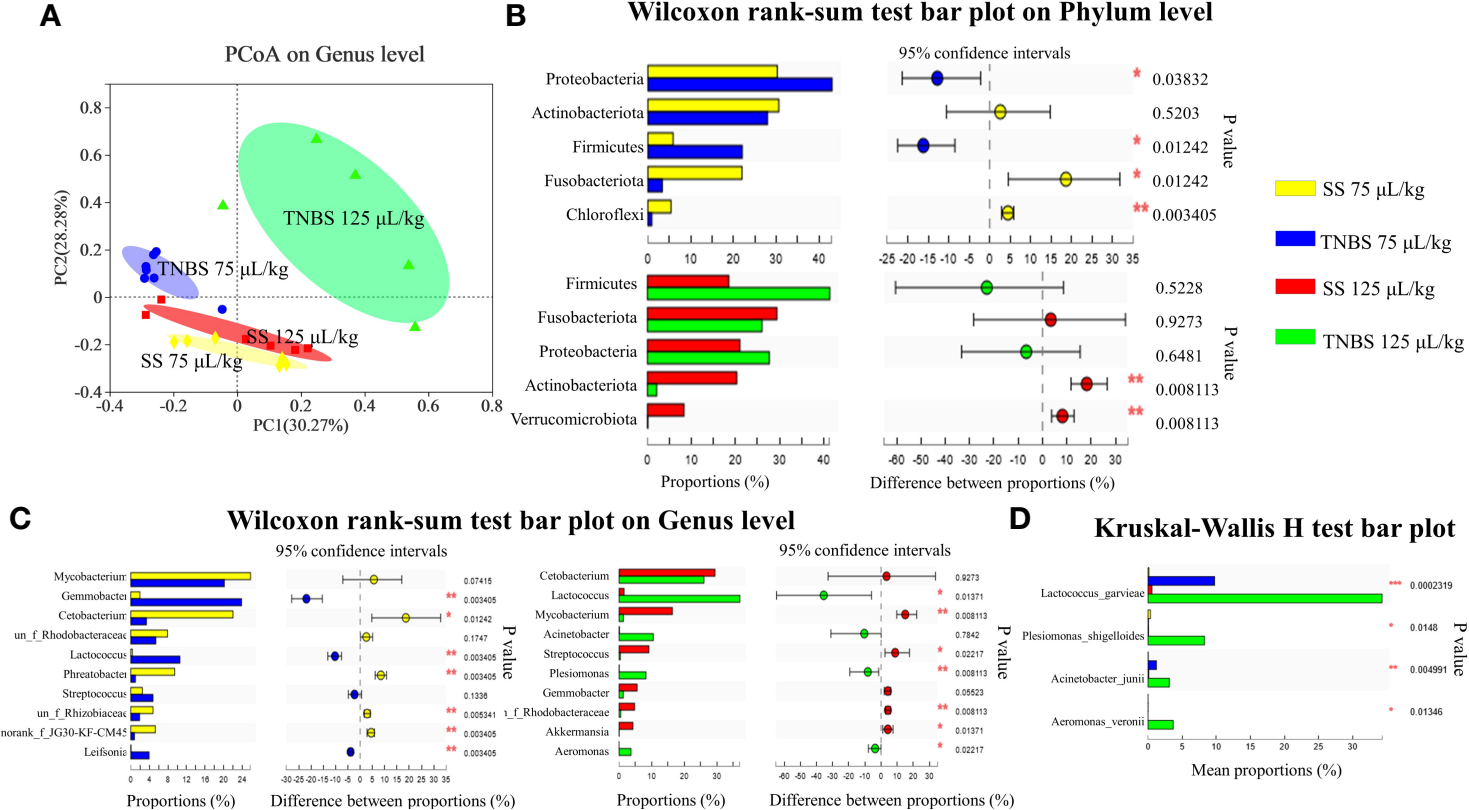
The effect of TNBS on the intestinal microbiota. **(A)** The principal co-ordinates analysis (PCoA) on genus level. **(B)** Relative abundance on Phylum level (top 5). **(C)** Relative abundance on Genu level (top 10). **(D)** Relative abundance of 4 pathogenic bacteria. N = 6 at least. *P* value was calculated by Student’s t tests. *P< 0.05, **P < 0.01, ***P < 0.001 compared with PBS groups.

Alpha diversity indices of the bacterial community showed that 125 μL/kg TNBS significantly decreased the community diversity and abundance. As shown in **Table 2**, the Shannon and Simpson diversity, and Chao and Ace richness were significantly affected in the 125 μL/kg TNBS group, but not in the 75 μL/kg TNBS group. Based on the distribution of samples on the plot, principal co-ordinates analysis (PCoA) at the genus level indicated differences between the four groups (**Figure 3A**).

**Table 2:** Alpha diversity of intestinal microbiota after treatment with SS and TNBS.

Treat	75 μL/kg SS	75 μL/kg TNBS	125 μL/kg SS	125 μL/kg TNBS
Sobs	471.38 ± 43.12	479.29 ± 73.47	486.57 ± 57.16	207 ± 174.46*
Shannon	3.01 ± 0.22	2.99 ± 0.44	3.00 ± 0.44	1.93 ± 0.97*
Simpson	0.14 ± 0.03	0.15 ± 0.07	0.16 ± 0.09	0.35 ± 0.23
Ace	721.91 ± 104.21	715.75 ± 124.03	722.39 ± 94.77	309.95 ± 276.35*
Chao	659.75 ± 80.58	641.67 ± 108.44	648.59 ± 68.72	289.76 ± 247.33*

*Indicate a significant difference in 125μL/kg TNBS compared with 125μL/kg SS groups.

Proteobacteria, Firmicutes, Actinobacteriota and Fusobacteriota were dominant phyla in the control groups (**Figure 3B**). However, 75 μL/kg TNBS significantly increased the relative abundances of Proteobacteria and Firmicutes phylum, and decreased the Fusobacteriota. In addition, 125 μL/kg TNBS increased Firmicutes while decreasing the Actinobacteriota.

At the genus level, *Mycobacterium* and *Cetobacterium* were the abundant genera in the control groups. A TNBS concentration of 75 μL/kg significantly decreased the abundance of *Cetobacterium* while increasing the abundance of *Gemmobacter* (**Figure 3C**). In addition, 125 μL/kg TNBS significantly increased the abundance of gram-negative bacteria, including *Acinetobacter*, *Plesiomonas*, and *Aeromonas* (**Figure 3C**). In particular, the abundance of *Lactococcus* significantly increased in TNBS-treated groups. We found that TNBS specifically increased the pathogenic bacteria *Lactococcus garvieae, Plesiomonas shigelloides, Acinetobacter junii and Aeromonas veronii* (**Figure 3D**).

### Effect of TNBS-induced enteritis on host injury parameters

**Figure 4:**
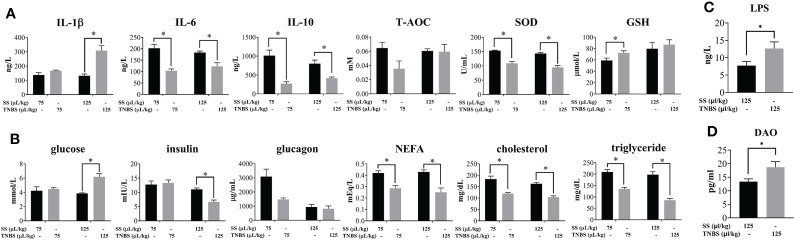
The effect of TNBS on the plasma inflammation, oxidative damage and glucolipid metabolism. **(A)** Plasma inflammatory cytokines, and T-AOC, SOD and GSH activity. **(B)** Plasma concentration of glucose, insulin, glucagon, NEFA, cholesterol and triglyceride. **(C)** Plasma concentration of LPS in 125μL/kg SS and 125μL/kg TNBS. **(D)** Plasma concentration of DAO in 125μL/kg SS and 125μL/kg TNBS. *P* value was calculated by Student’s t tests. All data are expressed as mean ± S.E.M. (N = 6 at least). *P* value was calculated by Student’s t tests. *P < 0.05, compared with PBS groups.

In order to evaluate whether acute enteritis induced by TNBS causes host injury, various biochemical indexes of plasma were analyzed. As shown in **Figure 4A**, IL-1β was significantly increased in the 125 μL/kg TNBS group, while the other cytokines (IL-6 and IL-10) were significantly decreased in the TNBS groups compared to the controls. The antioxidant biomarker SOD was significantly decreased in the TNBS groups, while GSH was significantly increased in the 75 μL/kg TNBS group.

Plasma glucose was significantly higher, while insulin was significantly decreased in the 125 μL/kg TNBS group compared to the control group (**Figure 4B**). In addition, both doses of TNBS significantly decreased the content of plasma lipids, including NEFA, cholesterol and triglycerides.

The increase in gut permeability led to the paracellular transport of luminal endotoxins into circulation. Compared to the SS groups, the level of LPS was significantly elevated in the 125 μL/kg TNBS group (**Figure 4C**). In agreement with the previous intestinal permeability results, the level of DAO in plasma was significantly higher in the 125 μL/kg TNBS group (**Figure 4D**).

### Metabolomic analysis of the liver

**Figure 5:**
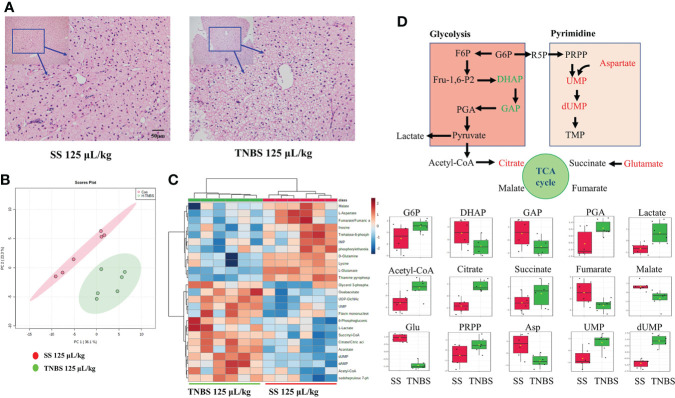
The effect of TNBS on the liver inflammation and energy metabolism. **(A)** The 25μL/kg SS and 125μL/kg TNBS representative microphotograph of H&E staining paraffin-embedded sections of liver was shown (scale bar: 50μm). **(B)** The PCA score plots of energy metabolism in two groups. **(C)** Heatmap of energy-related metabolites of two groups. **(D)** Schematic figure showing the energy metabolism, and box plots of the relative abundance of metabolites, red and green colors indicate significant up- and downregulation, respectively. N=6.

TNBS significantly influenced energy metabolism in gibel carp. In line with the changes in plasma biomarkers, histological analyses with H&E staining clearly revealed liver damage induced by TNBS, characterized by obvious macrovesicular steatosis and irregular arrangement of hepatocytes that was not seen in the control group (**Figure 5A**). To further explore the metabolic changes in the liver, the energy metabolism of liver samples was analyzed. The PCA result is shown in **Figure 5B**. Significant differences between the 125 μL/kg TNBS-treated groups and the SS groups were observed. Based on the top 25 metabolites, we also found that the heatmap plot clearly distinguished 125 μL/kg TNBS groups from the control groups (**Figure 5C**). **Figure 5D** shows the glycolysis metabolism, purine metabolism, pyrimidine metabolism as well as TCA and PPP pathways in the liver. Compared to the control group, changes in the metabolites of the group treated with 125 μL/kg TNBS were clearly visualized using boxplots. Compared to the control groups, the glyceraldehyde 3-phosphate, dihydroxyacetone phosphate, thiamine pyrophosphate (TPP) and aspartate were significantly decreased while the citrate and dUMP were significantly increased in 125 μL/kg TNBS groups.

### Liver respiration was affected in TNBS-induced enteritis of gibel carp

**Figure 6:**
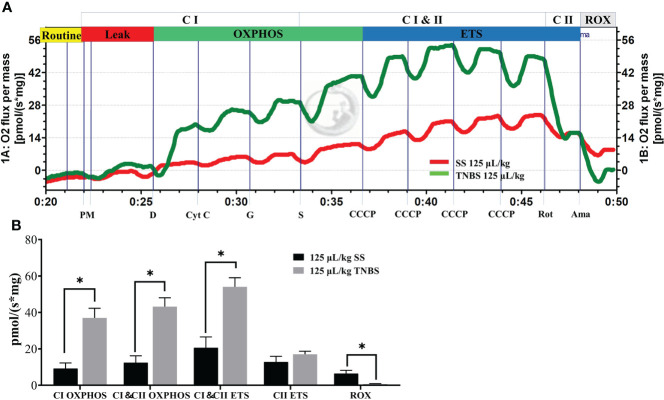
The TNBS enhanced the respiratory of mitochondria in liver. **(A)** Representative effects of TNBS on mitochondrial function. The oxygen flux at different respiration states is obtained by introducing specific substrates and inhibitors to the samples. Red line, oxygen consumption in response to the application of substrates for complex I (CI) and II (CII) with 125 μL/kg SS. Green line, with 125 μL/kg TNBS. **(B)** Summarized data for oxygen consumption in the liver as measured *via* Oxygraph-2k high-resolution respirometry. *P* value was calculated by Student’s t tests. All data are expressed as mean ± S.E.M. (N = 7). *P< 0.05, compared with PBS groups.

The substrate-uncoupler-inhibitor-titration (SUIT) approach together with high-resolution respirometry is able to assess the function of electron-transferring CI and CII, and ETS capacity (**Figures 6A, B**). Oxygen fluxes of the CI OXPHOS, as well as CI and CII OXPHOS (CI & CII OXPHOS) states were significantly higher than the control for the TNBS groups. A significant increase was also observed when the uncoupler (CCCP) was applied for the assessment of the maximal ETS (CI and CII ETS) capacity in 125 μL/kg TNBS groups. However, no differences in the oxygen fluxes of CII ETS states were observed after subsequent addition of CI inhibitor rotenone in HTNBS groups. Thus, 125 μL/kg TNBS significantly enhanced the CI OXPHOS, not CI OXPHOS capacity. At the end of the experiments, the resulting ROX in the 125 μL/kg TNBS groups was significantly lower than the control, demonstrating that the oxygen consumption was mainly of mitochondrial origin.

### Metabolomic analysis of the LPS treated NCTC1469 cells

**Figure 7:**
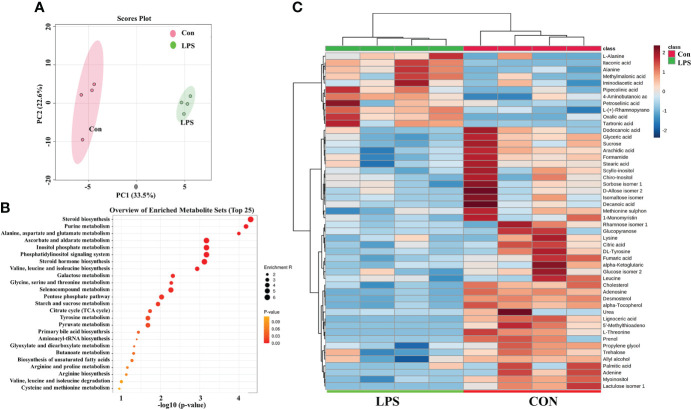
LPS affect the metabolism of liver cell line (NCTC-1469). **(A)** The principal components analysis (PCA) of two groups. **(B)** The metabolites enriched pathways (top 25) in PBS and LPS. **(C)** Heatmap of carbohydrate and lipid metabolites in PBS and LPS. N = 4.

The PCA result was shown in **Figure 7A** and it demonstrated significant differences between the LPS-treated group and the control (Con) group. An enriched chart of the metabolites provided a graphical display of the top 25 most enriched pathways (**Figure 7B**). The results showed that the most affected pathways were primarily related to amino acid and carbohydrate metabolism. The heatmap showed that most metabolites were downregulated in LPS groups while the itaconic acid was significantly upregulated (**Figure 7C**).

## Discussion

In the present study, we aimed to discover metabolic changes in the liver during enteritis. The results indicated that hepatic metabolism, in particular the upregulation of mitochondrial complex I, might be a compensatory or acute phase response against changes in intestinal inflammation. The intestinal microbiota and their endotoxins mediate this process.

Several intestinal inflammation models have been created in fish to clarify the mechanisms of inflammatory reactions and evaluate novel prevention methods, including soybean meal (16), pathogenic bacteria *Aeromonas hydrophila* (17), and chemical reagent TNBS (18). In the present study, the histological examination revealed that TNBS caused typical intestinal pathology, including villus atrophy, inflammatory cells infiltrating the lamina propria and disruption of the epithelial barrier as reported previously (16, 19).

The formation and permeability of tight junctions determine the resistance and integrity of the intestine (20). Dysregulation of tight junction proteins contributes to the pathogenesis of intestinal diseases (21). Meanwhile, intestinal inflammation affects the expression and cellular localization of TJ proteins, including the claudin family proteins, JAM and ZO. Some ultrastructural studies have demonstrated a dilation of TJ and increased intercellular space in mucosal biopsies of enteritis patients (22). Gene expression and ultrastructural examination in the current study revealed that tight junctions were disrupted after being treated with TNBS thereby confirming the ability of our model to simulate intestinal barrier disruption.

Alterations of intestinal epithelial homeostasis during inflammation may increase permeability of the gut barrier. Intestinal permeability was evaluated on the basis of the transepithelial electrical measurements with the Ussing chamber. The present study showed decreased TER revealing decreased physical intestinal barrier function which is in line with previous studies in Atlantic salmon (23). In addition, DAO is barely present in the circulation and its basal plasma levels are correlated with the integrity of the intestinal mucosa (24). In agreement with the TER results, the level of DAO in plasma was significantly elevated in TNBS groups compared to SS groups. In mammals, the cytokines (eg. IL-1β, IL-10, TNFα and TGFβ) mediate tight junction function (25). Thus, the barrier disruption in gibel carp may involve up-regulation of *il-1β* and *tgfβ* as observed in the current study.

As TNBS induced intestinal damage, high dosages may cause apoptosis of epithelial cells (26). The present study found that TUNEL staining was enhanced and the expression of apoptosis-related genes (*blc2* and *caspase*3) was significantly affected. Redox and oxidant-mediated regulation of apoptosis (27), and Nrf2 stimulates cytoprotective genes in response to oxidative stress both in fish and mammals and leads to activation of the downstream genes in Nrf2-Keap1 signaling pathway (28). We found that the gene expression of *nrf2*, *bach1* and *cat* was significantly down-regulated while the expression of *hsp70* was elevated after treatment with 125 μL/kg TNBS indicating that TNBS induced oxidative stress.

As a second factor associated with barrier function, intestinal microbiota was one of the factors responsible for enhancement of intestinal permeability (29). Several reports have shown that the changes in gut microbiota composition are associated with intestinal barrier function. In this study, we have clearly shown that the barrier disruption was associated with a significantly reduced gut microbiota diversity. In addition, numerous studies have found that an increase in the abundance of Proteobacteria reflects dysbiosis in gut microbiota (30) and plays a causative role in intestinal inflammation (31). Fusobacteriota metabolizes carbohydrates into butyrate which provides energy for gastrointestinal cells and has anti-inflammatory propoerties (31). Thus, in this study, the increased relative abundance of Proteobacteria and decreased relative abundance of Fusobacteriota had a detrimental effect on the maintenance of host intestinal energy supply and normal immuno homeostasis. Meanwhile, the genus level was significantly affected as well after treatment with TNBS. *Lactococcus garvieae, Plesiomonas shigelloides, Acinetobacter junii and Aeromonas veronii* are all pathogenic bacteria in aquaculture (32), and may cause gastroenteritis and septicemia (33, 34). The relative abundance of these bacteria increased significantly in the TNBS groups in a dose-dependent manner. Accordingly, TNBS-induced enteritis in gibel carp can be used as a model of intestinal inflammation.

With the development of enteritis, the intestinal barrier will be broken and bacteria and bacterial endotoxins will enter circulation. In response to enteritis, the intestinal epithelium changes structurally, allowing LPS to enter the bloodstream, resulting in an increase in the plasma levels of LPS (35). LPS activates Toll-like receptor-4 (TLR4) leading to the production of pro-inflammatory cytokines and systemic inflammation (35). Our results showed that intestinal inflammation increases plasma LPS, and leads to liver damage. The same result was found in the SBM-induced intestinal inflammation model (16).

In agreement with many other studies (10, 36–38), our study indicated that dysfunction of energy metabolism is involved in intestinal inflammation. The increased plasma glucose and decreased NEFA, cholesterol, and triglyceride in the present study suggest glycolipid metabolism may be altered in response to intestinal inflammation. Zebeli et al. found that an oral LPS challenge significantly increased plasma glucose and decreased the plasma concentration of NEFA in cows (39). This suggests that the LPS translocation may contribute to the dysfunction of metabolism.

The energy metabolic analysis provides comprehensive information on central carbon metabolism-related changes. In the present study, we analyzed the influence of high dose TNBS with a more obvious phenotype on metabolism. The results showed that changes in glycolysis, the TCA cycle and pyrimidine metabolism occurred in the liver in response to severe intestinal inflammation after treatment with 125 μL/kg TNBS. Similarly, Kim et al. found that the levels of liver pyrimidine metabolism-related metabolites (uracil, UMP and UDP) increased significantly in the state of DSS-induced gut inflammation. These metabolites are associated with the synthesis of nucleotides and are important in the anti-inflammatory response and in preventing DNA damage (13). Although altered metabolism can confer several advantages with respect to inflammation, cells may be burdened with toxic byproducts as a consequence of a deranged or simply overactive metabolism (40). The level of glycolysis-related metabolites (DHAP and GAP) decreased significantly, whereas G6P, PGA and lactate increased in TNBS groups, suggesting enhanced glycolysis to lactate production. The elevated levels of citric acid in the TCA cycle revealed that, in addition to glycolysis, lipolysis may also be enhanced in the TNBS groups. In addition, succinate has been identified as an inflammatory signal and glutamine-dependent anaplerosis is the principal source of succinate during inflammation (41). Thus, the significantly higher levels of succinate and glutamate indicated that the liver actively responded to inflammation.

Some intermediates of central carbon metabolism are involved in the OXPHOS. Inflammation is an energy-intensive process and the mitochondria play crucial roles in supporting these energy-requiring responses. The colonic mucosa in enteritis is in a state of energy deficiency characterized by low energy charge and low adenosine triphosphate (ATP) levels. Herein, we found mitochondrial abnormalities in the liver as well. However, different from the gut, the activity of Complex I (NADH: ubiquinone oxidoreductase) was significantly promoted in liver. We observed that enteritis increased the respiratory function in liver at the CI & CII OXPHOS, and CI & CII ETC states. Our results demonstrate that liver increased ATP synthase activity and enhanced mitochondria oxidative phosphorylation during enteritis. The site for ROS production in mitochondria is normally ascribed to the activity of Complex I (42) which accepts electrons from nicotinamide adenine dinucleotide (NAD+) generated in the TCA. Thus, active mitochondria in the liver produce high levels of ATP as a compensatory response to the acute challenge of TNBS-induced inflammation. However, in a high metabolic state, the liver also produces more ROS which would damage the mitochondria and impede the production of high ATP levels. Some researchers have found that mitochondrial respiratory chain complex I inhibitors (rotenone and metformin) can alleviate the LPS-induced fulminant liver injury (43, 44). Thus, mitochondrial respiratory chain complex I may potentially be involved in the control of TNBS-induced inflammation.

We further confirmed that LPS induces disturbances in liver metabolism. Most studies focused on the metabolic changes in macrophages after LPS treatment. The *in vitro* research has demonstrated that upon LPS stimulation, the hepatic macrophages activate to release the inflammatory cytokines (45). The activated macrophages shift from producing ATP by oxidative phosphorylation to aerobic glycolysis. In the present study, we analyzed the metabolome of NCTC1469 cells after LPS treatment and demonstrated that LPS significantly affects the metabolism of hepatocytes, not only the macrophages. Thus, we speculated that intestinal inflammation directly affected liver metabolism.

## Conclusion

In conclusion, we developed an intestinal inflammation model in gibel carp with TNBS. We found that gut microbiota-derived endotoxins mediated changes in liver metabolism, especially the activity of Complex I in response to inflammation. Our results further indicate that intestinal inflammation results in dysbiosis of microbiota and leads to the production of a large number of endotoxic LPS which enter the circulation. LPS induced the activation of mitochondria to enhance energy production. Thus, targeting the energy biosynthetic pathways in the liver might be a novel strategy for the therapy of systemic inflammation during enteritis.

## Data availability statement

The datasets presented in this study can be found in online repositories. The names of the repository/repositories and accession number(s) can be found below: NCBI: PRJNA856173, MetaboLights: MTBLS5270.

## Ethics statement

The animal study was reviewed and approved by Institute of Hydrobiology, Chinese Academy of Sciences.

## Author contributions

LF: Investigation, Formal analysis, Data curation, Writing- Original draft preparation. HL: Conceptualization, Data curation, Project administration, Writing - review & editing. WC: Investigation. JH and MØ: Review and editing. WJC: Investigation. DH, XZ, and JJ: Methodology. YY: Resources. SX: Supervision, Funding acquisition. All authors contributed to the article and approved the submitted version.

## Funding

This work was financially supported by the earmarked fund for China Agriculture Research System of MOF and MARA (CARS-45), the National Key R&D Program of China (2019YFD0900200), and National Natural Science Foundation of China (31602174).

## Acknowledgments

The authors thank Ms. Yanxia Zuo and Yuan Xiao (the Center for Instrumental Analysis and Metrology, Institute of Hydrobiology, Chinese Academy of Science) for their technical assistance in GC-MS analysis and Transmission electron microscopy observation. The authors also thank Mr. Guanghan Nie for his technical assistance.

## Conflict of interest

The authors declare that the research was conducted in the absence of any commercial or financial relationships that could be construed as a potential conflict of interest.

## Publisher’s note

All claims expressed in this article are solely those of the authors and do not necessarily represent those of their affiliated organizations, or those of the publisher, the editors and the reviewers. Any product that may be evaluated in this article, or claim that may be made by its manufacturer, is not guaranteed or endorsed by the publisher.

## References

[1] Chassaing B Aitken JD Malleshappa M Vijay-Kumar M. Dextran sulfate sodium (DSS)-induced colitis in mice. Curr Protoc Immunol (2014) 104:15 25 1–15 25 14. doi: 10.1002/0471142735.im1525s104 24510619 PMC3980572

[2] Baumgart DC Sandborn WJ. Crohn's disease. Lancet (2012) 380(9853):1590–605. doi: 10.1016/s0140-6736(12)60026-9 22914295

[3] Xavier RJ Podolsky DK. Unravelling the pathogenesis of inflammatory bowel disease. Nature (2007) 448(7152):427–34. doi: 10.1038/nature06005 17653185

[4] Teirlynck E Bjerrum L Eeckhaut V Huygebaert G Pasmans F Haesebrouck F. The cereal type in feed influences gut wall morphology and intestinal immune cell infiltration in broiler chickens. Br J Nutr (2009) 102(10):1453–61. doi: 10.1017/S0007114509990407 19664304

[5] Huang SM Wu ZH Li TT Liu C Han DD Tao SY. Perturbation of the lipid metabolism and intestinal inflammation in growing pigs with low birth weight is associated with the alterations of gut microbiota. Sci Total Environ (2020) 719:137382. doi: 10.1016/j.scitotenv.2020.137382 32114228

[6] Wang J Liang D Yang Q Tan B Dong X Chi S. The effect of partial replacement of fish meal by soy protein concentrate on growth performance, immune responses, gut morphology and intestinal inflammation for juvenile hybrid grouper (*Epinephelus fuscoguttatus* ♀× *Epinephelus lanceolatus*♂). Fish Shellfish Immunol (2020) 98:619–31. doi: 10.1016/j.fsi.2019.10.025 31704202

[7] Wu N Xu X Wang B Li XM Cheng YY Li M. Anti-foodborne enteritis effect of galantamine potentially *via* acetylcholine anti-inflammatory pathway in fish. Fish Shellfish Immunol (2020) 97:204–15. doi: 10.1016/j.fsi.2019.12.028 31843701

[8] Ma N Guo P Zhang J He T Kim SW Zhang G. Nutrients mediate intestinal bacteria-mucosal immune crosstalk. Front Immunol (2018) 9:5. doi: 10.3389/fimmu.2018.00005 29416535 PMC5787545

[9] Al Bander Z Nitert MD Mousa A Naderpoor N. The gut microbiota and inflammation: An overview. Int J Environ Res Public Health (2020) 17(20):7618. doi: 10.3390/ijerph17207618 PMC7589951 33086688

[10] Bar F Bochmann W Widok A von Medem K Pagel R Hirose M. Mitochondrial gene polymorphisms that protect mice from colitis. Gastroenterology (2013) 145(5):1055–63 e3. doi: 10.1053/j.gastro.2013.07.015 23872498

[11] Sifroni KG Damiani CR Stoffel C Cardoso MR Ferreira GK Jeremias IC. Mitochondrial respiratory chain in the colonic mucosal of patients with ulcerative colitis. Mol Cell Biochem (2010) 342(1-2):111–5. doi: 10.1007/s11010-010-0474-x 20440543

[12] Sartini A Gitto S Bianchini M Verga MC Di Girolamo M Bertani A. Non-alcoholic fatty liver disease phenotypes in patients with inflammatory bowel disease. Cell Death Dis (2018) 9(2):87. doi: 10.1038/s41419-017-0124-2 29367619 PMC5833704

[13] Kim SH Lee W Kwon D Lee S Son SW Seo MS. Metabolomic analysis of the liver of a dextran sodium sulfate-induced acute colitis mouse model: Implications of the gut-liver connection. Cells (2020) 9(2). doi: 10.3390/cells9020341 PMC7072179 32024178

[14] Liu H Jin J Zhu X Han D Yang Y Xie S. Effect of substitution of dietary fish meal by soybean meal on different sizes of gibel carp (*Carassius auratus gibelio*): Digestive enzyme gene expressions and activities, and intestinal and hepatic histology. Aquacult Nutr (2017) 23(1):129–47. doi: 10.1111/anu.12375

[15] Clarke LL. A guide to ussing chamber studies of mouse intestine. Am J Physiol Gastrointest Liver Physiol (2009) 296(6):G1151–66. doi: 10.1152/ajpgi.90649.2008 PMC2697950 19342508

[16] Fu L Liu H Cai W Han D Zhu X Yang Y. 4-octyl itaconate supplementation relieves soybean diet-induced liver inflammation and glycolipid metabolic disorders by activating the Nrf2-Pparϒ pathway in juvenile gibel carp. J Agric Food Chem (2022) 70(2):520–31. doi: 10.1021/acs.jafc.1c05783 34881880

[17] Song X Zhao J Bo Y Liu Z Wu K Gong C. Aeromonas hydrophila induces intestinal inflammation in grass carp (*Ctenopharyngodon idella*): An experimental model. Aquaculture (2014) 434:171–8. doi: 10.1016/j.aquaculture.2014.08.015

[18] Oehlers SH Flores MV Hall CJ Okuda KS Sison JO Crosier KE. Chemically induced intestinal damage models in zebrafish larvae. Zebrafish (2013) 10(2):184–93. doi: 10.1089/zeb.2012.0824 23448252

[19] Xie J Li M Ye W Shan J Zhao X Duan Y. Sinomenine hydrochloride ameliorates fish foodborne enteritis *via* Alpha7nachr-mediated anti-inflammatory effect whilst altering microbiota composition. Front Immunol (2021) 12:766845. doi: 10.3389/fimmu.2021.766845 34887862 PMC8650311

[20] Li H Sheppard DN Hug MJ. Transepithelial electrical measurements with the ussing chamber. J Cyst Fibros (2004) 3 Suppl 2:123–6. doi: 10.1016/j.jcf.2004.05.026 15463943

[21] Martinez C Lobo B Pigrau M Ramos L Gonzalez-Castro AM Alonso C. Diarrhoea-predominant irritable bowel syndrome: An organic disorder with structural abnormalities in the jejunal epithelial barrier. Gut (2013) 62(8):1160–8. doi: 10.1136/gutjnl-2012-302093 22637702

[22] Lechuga S Ivanov AI. Disruption of the epithelial barrier during intestinal inflammation: Quest for new molecules and mechanisms. Biochim Biophys Acta Mol Cell Res (2017) 1864(7):1183–94. doi: 10.1016/j.bbamcr.2017.03.007 PMC5507344 28322932

[23] Knudsen D Jutfelt F Sundh H Sundell K Koppe W Frokiaer H. Dietary soya saponins increase gut permeability and play a key role in the onset of soyabean-induced enteritis in Atlantic salmon ( *Salmo salar l.*). Br J Nutr (2008) 100(1):120–9. doi: 10.1017/S0007114507886338 18167174

[24] Wolvekamp MC de Bruin RW. Diamine oxidase: An overview of historical, biochemical and functional aspects. Dig Dis (1994) 12(1):2–14. doi: 10.1159/000171432 8200121

[25] Capaldo CT Nusrat A. Cytokine regulation of tight junctions. Biochim Biophys Acta (2009) 1788(4):864–71. doi: 10.1016/j.bbamem.2008.08.027 PMC2699410 18952050

[26] Antoniou E Margonis GA Angelou A Pikouli A Argiri P Karavokyros I. The TNBS-induced colitis animal model: An overview. Ann Med Surg (Lond) (2016) 11:9–15. doi: 10.1016/j.amsu.2016.07.019 27656280 PMC5021709

[27] Haddad JJ. Redox and oxidant-mediated regulation of apoptosis signaling pathways: Immuno-Pharmaco-Redox conception of oxidative siege versus cell death commitment. Int Immunopharmacol (2004) 4(4):475–93. doi: 10.1016/j.intimp.2004.02.002 15099526

[28] Ma Q. Role of Nrf2 in oxidative stress and toxicity. Annu Rev Pharmacol Toxicol (2013) 53:401–26. doi: 10.1146/annurev-pharmtox-011112-140320 PMC4680839 23294312

[29] Takashima S Tanaka F Kawaguchi Y Usui Y Fujimoto K Nadatani Y. Proton pump inhibitors enhance intestinal permeability *via* dysbiosis of gut microbiota under stressed conditions in mice. Neurogastroenterol Motil (2020) 32(7):e13841. doi: 10.1111/nmo.13841 32319196

[30] Shin NR Whon TW Bae JW. Proteobacteria: Microbial signature of dysbiosis in gut microbiota. Trends Biotechnol (2015) 33(9):496–503. doi: 10.1016/j.tibtech.2015.06.011 26210164

[31] Zhang J Meng H Kong X Cheng X Ma T He H. Combined effects of polyethylene and organic contaminant on zebrafish (*Danio rerio*): Accumulation of 9-nitroanthracene, biomarkers and intestinal microbiota. Environ Pollut (2021) 277:116767. doi: 10.1016/j.envpol.2021.116767 33640823

[32] Gong L He H Li D Cao L Khan TA Li Y. A new isolate of *Pediococcus pentosaceus* (Sl001) with antibacterial activity against fish pathogens and potency in facilitating the immunity and growth performance of grass carps. Front Microbiol (2019) 10:1384. doi: 10.3389/fmicb.2019.01384 31316478 PMC6610308

[33] Shahi N Mallik SK. Emerging bacterial fish pathogen *Lactococcus garvieae* Rtcli04, isolated from rainbow trout (*Oncorhynchus mykiss*): Genomic features and comparative genomics. Microb Pathog (2020) 147:104368. doi: 10.1016/j.micpath.2020.104368 32634612

[34] Cortes-Sanchez AJ Espinosa-Chaurand LD Diaz-Ramirez M Torres-Ochoa E. *Plesiomonas*: A review on food safety, fish-borne diseases, and tilapia. Sci World J (2021) 2021:3119958. doi: 10.1155/2021/3119958 PMC8478591 34594160

[35] Mohammad S Thiemermann C. Role of metabolic endotoxemia in systemic inflammation and potential interventions. Front Immunol (2020) 11:594150. doi: 10.3389/fimmu.2020.594150 33505393 PMC7829348

[36] Nong F Luo S Liang Y Zhao Z Xing S Wen B. Evaluation of the effect of dahuang-mudan decoction on tnbs-induced colitis using uplc-Qtof/Ms-Based metabolomic analysis. BioMed Chromatogr (2021) 35(3):e5003. doi: 10.1002/bmc.5003 33063880

[37] Fukushima K Fiocchi C. Paradoxical decrease of mitochondrial DNA deletions in epithelial cells of active ulcerative colitis patients. Am J Physiol Gastrointest (2004) 286(5):G804–813. doi: 10.1152/ajpgi.00398.2003 15068964

[38] Jiang XG Sun K Liu YY Yan L Wang MX Fan JY. Astragaloside iv ameliorates 2,4,6-trinitrobenzene sulfonic acid (TNBS)-induced colitis implicating regulation of energy metabolism. Sci Rep (2017) 7:41832. doi: 10.1038/srep41832 28150820 PMC5288804

[39] Zebeli Q Mansmann D Sivaraman S Dunn SM Ametaj BN. Oral challenge with increasing doses of lps modulated the patterns of plasma metabolites and minerals in periparturient dairy cows. Innate Immun (2013) 19(3):298–314. doi: 10.1177/1753425912461287 23109506

[40] Hsu PP Sabatini DM. Cancer cell metabolism: Warburg and beyond. Cell (2008) 134(5):703–7. doi: 10.1016/j.cell.2008.08.021 18775299

[41] Tannahill GM Curtis AM Adamik J Palsson-McDermott EM McGettrick AF Goel G. Succinate is an inflammatory signal that induces IL-1β through HIF-1α. Nature (2013) 496(7444):238–42. doi: 10.1038/nature11986 PMC4031686 23535595

[42] Fato R Bergamini C Leoni S Lenaz G. Mitochondrial production of reactive oxygen species: Role of complex I and quinone analogues. Biofactors (2008) 32(1-4):31–9. doi: 10.1002/biof.5520320105 19096098

[43] Ai Q Jing Y Jiang R Lin L Dai J Che Q. Rotenone, a mitochondrial respiratory complex I inhibitor, ameliorates Lipopolysaccharide/D-Galactosamine-Induced fulminant hepatitis in mice. Int Immunopharmacol (2014) 21(1):200–7. doi: 10.1016/j.intimp.2014.04.028 24830863

[44] Yuan H Li L Zheng W Wan J Ge P Li H. Antidiabetic drug metformin alleviates endotoxin-induced fulminant liver injury in mice. Int Immunopharmacol (2012) 12(4):682–8. doi: 10.1016/j.intimp.2012.01.015 22330083

[45] Wang R Tang R Li B Ma X Schnabl B Tilg H. Gut microbiome, liver immunology, and liver diseases. Cell Mol Immunol (2021) 18(1):4–17. doi: 10.1038/s41423-020-00592-6 33318628 PMC7852541

